# Viral oncomiR spreading between B and T cells is employed by Kaposi's sarcoma herpesvirus to induce non-cell-autonomous target gene regulation

**DOI:** 10.18632/oncotarget.9627

**Published:** 2016-05-26

**Authors:** Nir Rainy, Morad Zayoud, Yoel Kloog, Oded Rechavi, Itamar Goldstein

**Affiliations:** ^1^ Sheba Cancer Research Center, Chaim Sheba Academic Medical Center, Tel Hashomer, Ramat Gan 5262100, Israel; ^2^ Sackler School of Medicine, Tel-Aviv University, Tel-Aviv 6997801, Israel; ^3^ Department of Neurobiology, The George S. Wise Faculty of Life Sciences & Sagol School of Neuroscience, Tel-Aviv University, Tel-Aviv 6997801, Israel

**Keywords:** microRNAs, herpesviruses, lymphoma, oncomiRs, viral carcinogenesis

## Abstract

The two human lymphotrophic γ-herpesviruses, Kaposi's sarcoma herpesvirus (KSHV) and Epstein-Barr virus (EBV), are a recognized cause of human cancer, encoding multiple miRs that are major players in carcinogenesis. Previously, we discovered that EBV-encoded miRs transfer between infected B and T lymphocytes. To further explore the biological significance of the spreading of γ-herpesvirus-encoded miRs on carcinogenesis, we focused on KSHV-miR-K12-11 (miR-K12-11) that is unique in having an identical seed sequence with the oncomiR *hsa*-miR-155, implicated in B cell lymphomas development. Here, we show for the first time that miR-K12-11 transfers *in vitro* from KSHV-infected BCBL-1 and BC-1 lymphoma lines to T cells. The transferred miR-K12-11 is active in the adopting T cells and binds its canonical target, the 3′-UTR of BACH1. Importantly, we show that the transfer of miR-K12-11 from BCBL-1 to Jurkat cells correlates with inhibition of the innate type-I interferons response to viral dsRNAs downstream of IKKε, a validated miR-K12-11 target. Finally, we show that miR-K12-11 spreading is not reduced by blocking the classical ceramide-dependent exosome secretion pathway. In summary, we report for the first time that intercellular viral oncomiR spreading is an additional mechanism employed by KSHV to inhibit host anti-viral immunity and consequently promote oncogenesis.

## INTRODUCTION

The viral agents Epstein-Barr virus (EBV) and Kaposi's sarcoma-associated herpesvirus (KSHV) are the two lymphotrophic γ-herpesviruses that have evolved specifically to maintain persistent latent infections in humans. There is substantial evidence from biomedical research that implicates γ-herpesviruses in the development of human cancer, primarily B cell malignancies [1, 2].

Latent infection with γ-herpesviruses are associated with the expression of non-coding RNAs from the viral genome, including miR precursors predicted to target many host genes [3]. The KSHV and EBV genomes contains 12 and 25 validated miR loci, respectively, which encode for multiple mature miRs [3]. Although, EBV- and KSHV-encoded miRs do not share significant sequence homology they target a large number of similar genes [4].

In plants and invertebrates small non coding dsRNAs have been shown to spread systemically and transmit molecular information such as gene silencing [4]. Moreover, in Caenorhabditis Elegans small dsRNAs have also been shown to mediate transgenerational epigenetic inheritance of parental responses to past conditions such as viral infection [5] and starvation [6]. Although extracellular small RNA species, chiefly miRs, have been identified in mammalian body fluids, the biological roles of these mobile small ncRNAs are not well-defined [7]. Since the report by Valadi et al. that exosomes secreted from immune cells can serve as transporters of extracellular miRs [8] there has been a mounting interest in the study of miR spreading among mammalian cells. In a previous study, we demonstrated that short dsRNAs transfer efficiently among B and T lymphocytes and that this transfer is augmented by immunological synapse formation [9]. We further discovered that certain EBV-encoded miRs transfer from Raji cells (EBV-infected lymphoma cells) into T cells during co-culturing [9] and this observation was subsequently confirmed *in vivo* [10]. To further explore the biological relevance of this mode of virus-host interaction, we focused on KSHV-miR-K12-11 (miR-K12-11), unique among the γ-herpesviruses miRs in having an identical seed sequence with *hsa*-miR-155, and thus functions as its viral orthologue targeting similar transcripts [11]. MiR-155 is an evolutionary conserved multifunctional miR that has been widely studied and is important in immunity and lymphocyte development [12]. Moreover, it has been classified as an oncomiR implicated in promoting the development of various hematological and solid malignancies [13].

In this study, using an *in vitro* co-culture system we determined that the viral oncogenic miR-K12-11 spreads into the extra cellular environment and shuttles into T cells, where it can reduce target gene expression and repress the IKKε-dependent innate response to dsRNAs in a non-cell-autonomous mode.

## RESULTS

### BC-1 and BCBL-1 cells produce miR-K12-11 and transfer synthetic scrambled miRs to Jurkat T cells

**Figure 1:**
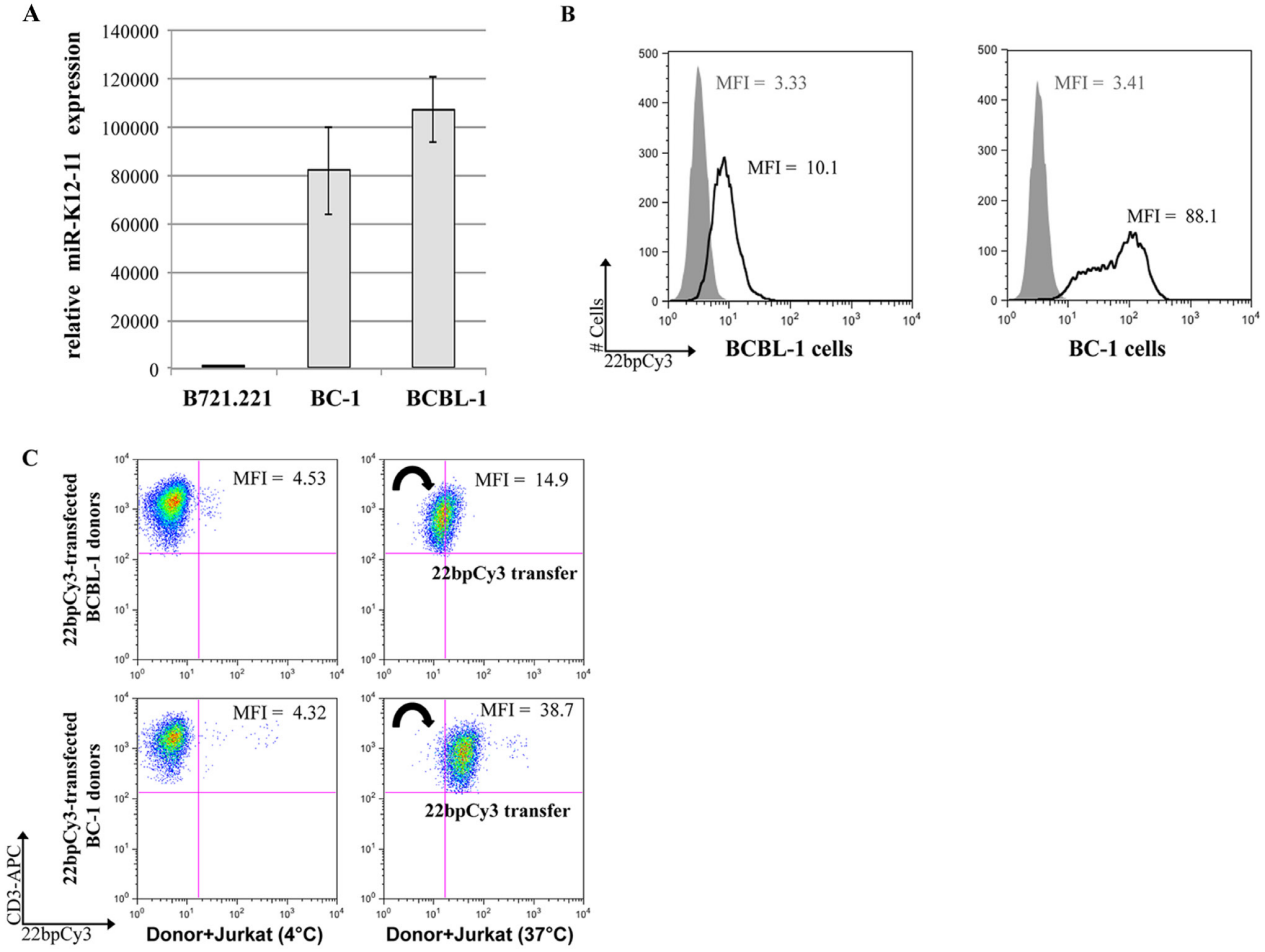
BC-1 cells express high levels of miR-K12-11 and efficiently spread miRs to T cells **A.** Relative expression (fold change) of miR-K12-11 in KSHV-infected BC-1 and BCBL-1 compared to the uninfected B721.221 cells by qRTPCR. Bars represent mean ± S.E.M. **B.** BC-1 and BCBL-1 cells were transfected by electroporation with 22bpCy3 scrambled synthetic miRs, and transfection efficiency was determined by FACS. The histogram represent 22bpCy3 (black line) versus background fluorescence (grey shade). **C.** Jurkat T cells were co-cultured with 22bpCy3-transfected BCBL-1 (upper panels) or BC-1 (lower panels) cells for 3 hour to test their ability to transfer synthetic miRs. Acquisition of 22bpCy3 by the Jurkat cells was then determined by FACS. Plots depict 22bpCy3 content in Jurkat cells (CD3^+^ gate only) from the various experimental groups after 3 hours of co-culturing. The data was acquired on a FACSCalibur instrument (~10,000 single cell events) and analyzed using FlowJo V.10 software. The results shown represent a typical experiment out of >5 performed.

KSHV-infected B lymphoma cell lines generally express latency associated viral transcripts including viral miRs [14]. While BC-1 is dually-infected with KSHV and EBV [15], BCBL-1 is infected by KSHV alone [16]. Initially we tested the expression levels of miR-K12-11, the oncogenic *hsa*-miR-155 orthologue, in these two cell-lines by qRTPCR. We found that both BCBL-1 and BC-1 cells expressed very high levels of the mature miR-K12-11 normalized to the KSHV-free/EBV-infected B721.221 cells (Figure 1A). We next examined the potential of BCBL-1 and BC-1 cells to transfer miRs to T cells by a FACS based method previously established by our group [9]. Thus, both BC-1 and BCBL-1 cells were transfected with the “scrambled” synthetic miR mimetic 22bpCy3 (Figures 1B). Twenty-four hours post-transfection the cells were washed thoroughly to remove surplus 22bpCy3 from the media, and then co-cultured for 3 hours with Jurkat cells. The cells were harvested and immunostained with labeled anti-CD3 mAbs. Acquisition of 22bpCy3 by CD3^+^ Jurkat cells was measured by FACS, using a stringent doublet (cell fusion) discrimination algorithm as previously described [9, 17]. We found that 22bpCy3 transferred efficiently from BC-1 and BCBL-1 to Jurkat cells, implying that both donor cells have the capacity to spread miRs.

### KSHV-miR-K12-11 transfers from BC-1 cells to Jurkat T cells

**Figure 2:**
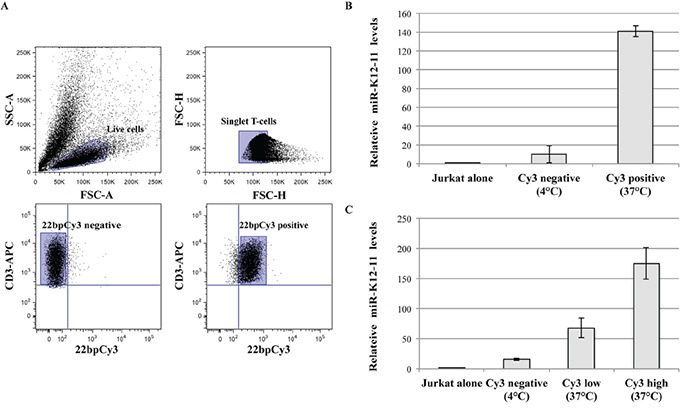
miR-K12-11 transfer correlated with 22bpCy3 transfer during co-culturing **A.** In these experiments 22bpCy3 served as a marker for miR transfer. The plots illustrate the sorting strategy and post-sorting purity analysis: (i) Pre-sort live cell gate based on side scatter area (SSC-A) and forward scatter area (FSC-A) features. (ii) Pre-sort singlet lymphocyte gate as defined by FSC-H (height) and FSC-W (width) parameters. (iii) Post-sort purity analysis (4°C co-cultures) for Cy3^neg^ singlet CD3^+^ Jurkat cells. (iv) Post-sort purity analysis (37°C co-cultures) for Cy3^pos^ singlet CD3^+^ Jurkat cells from, using a similar gating strategy for both. **B.** Relative levels of the transferred miR-K12-11 in isolated Cy3^pos^ and Cy3^neg^ cells and control Jurkat cells, as determined by qRTPCR and calculated using the 2^−ΔΔCt^ method normalized to RNU6B. **C.** Similar set up as above, but we also sorted out Cy3^high^, Cy3^low^, and Cy3^neg^ Jurkat cells and determined relative miR-K12-11 levels as above by qRTPCR. Bars represent fold change (mean ± S.E.M of duplicates) of a representative experiments out of >3 performed.

Next, we asked whether miR-K12-11 can also, similarly, transfer from BC-1 to T cells. Thus, BC-1 cells were transfected with 22bpCy3 and co-cultured with Jurkat cells. Three hours later, using a FACSAria instrument we sorted out from the co-cultures a highly pure (>95%) population of CD3^+^ 22bpCy3^pos+^ singlet Jurkat cells, as previously described [9]. Since endogenous and synthetic miR transfer is minimal at 4°C [9], we also sorted out Jurkat cells from co-cultures maintained at 4°C as an additional control (Figure 2A). The RNA isolated from the different sorted Jurkat populations and pure Jurkat cells was analyzed for miR-K12-11 by qRTPCR as detailed in methods. We found a >120-fold change in mobile miR-K12-11 levels in Cy3^pos^ Jurkat cells isolated from standard 37°C co-cultures compared to reference control Jurkat cells. As predicted, in the Cy3^neg^ Jurkat cells isolated from control 4°C cultures we detected a substantially smaller levels of miR-K12-11. Moreover, in supplementary experiments we further sorted out the Jurkat cells from standard 37°C co-cultures into Cy3^low^ and Cy3^hi^ fractions, and found a positive correlation between the relative quantity of 22bpCy3 and of miR-K12-11 acquired by T cells from donor BC-1 cells (Figure 2C). These data demonstrate that a KSHV-encoded oncomiR, produced by the classic cellular miR biogenesis pathway, transfers to neighboring T cells in an active temperature-sensitive cellular mechanism analogous to the spreading of synthetic exogenous miRs.

### Mature immunological synapse (IS) formation is not essential for miR spreading

**Figure 3:**
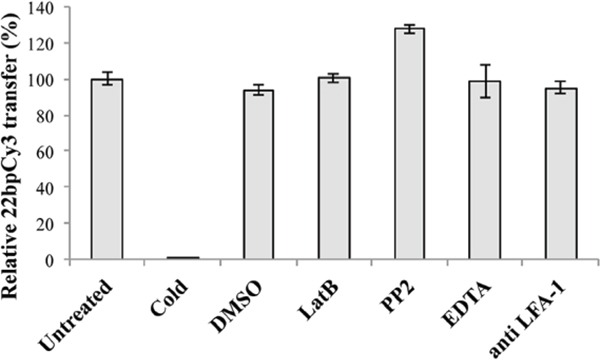
The intercellular transfer of miRs in independent of mature immunological synapse formation Jurkat cells and 22bpCy3-BC-1 cells were treated separately with the indicated inhibitor or left untreated. Bars chart represents the relative percentage of 22bpCy3 transfer detected for the various Jurkat cell treatment groups compared to control untreated cells (the latter condition set as 100% transfer). Data are from a typical experiment out of >3 performed in duplicates. Bars represent mean ± S.E.M, and p<0.01 for all groups (comparing the raw 22bpCy3 fluorescence intensity values by paired Student's t-test).

After demonstrating that γ-herpesviruses can exploit cellular pathways to spread viral miRs, we aimed to better elucidate the cellular mechanism(s) involved in this process. Previous reports imply that IS formation among lymphocytes and their targets may enhance intercellular spreading of host miRs [18]. To determine the role of IS formation on this transfer, we tested whether treatment with the actin polymerization inhibitor LatB, known to inhibit mature IS formation [19], reduces the intercellular spreading of 22bpCy3. Thus, BC-1 cells transfected by electroporation with 22bpCy3 were co-cultured with Jurkat cells for 3 hours in the presence of the LatB. In parallel, we also treated corresponding co-cultures with other compounds known to interfere by other mechanisms with the formation of a mature functional IS, such as: EDTA [19], anti-LFA1 blocking mAbs [20], and the Src kinase inhibitor PP2 [21]. We found that treatment with this range of validated inhibitors of IS formation did not hinder the transfer of 22bpCy3 into Jurkat cells as opposed to co-culturing in 4°C cold medium (Figure 3).

### Synthetic and viral miR spreading is dependent on secretory pathways

**Figure 4:**
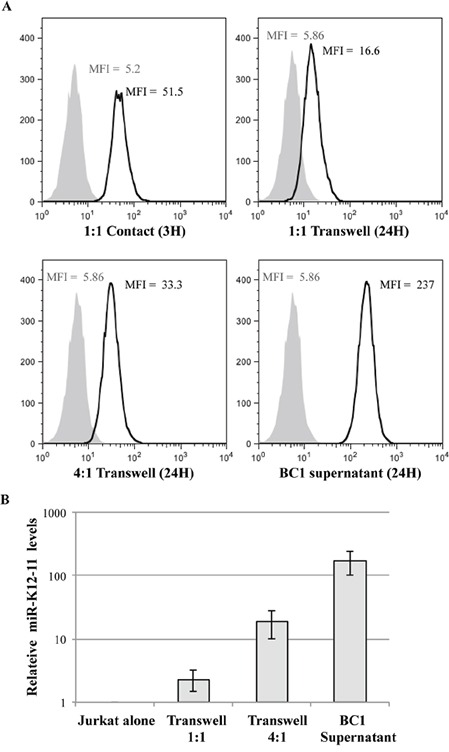
Synthetic scrambled and viral miRs are co-mobile via a secretory pathway **A.** Jurkat cells were co-cultured for 3 hours in contact with 22bpCy3-BC-1 cells (left upper panel), or for 24 hours with 22bpCy3-BC-1 cells separated by transwell membrane (in donor-to-acceptor ratio of 1:1 or 4:1) or in BC-1 filtered supernatant. The histograms represent the level of 22bpCy3 in the adopting Jurkat cells (black line) compared to background fluorescence (grey shade). **B.** At the end of co-culturing in the transwell system total RNA was isolated from the various cell cultures for qRTPCR analysis. Bar chart (*y*-axis in logarithmic scale) represents the fold-chance of mir-K12-11 (mean ± S.E.M of duplicates) in the various Jurkat groups compared to control cells. Depicted is a typical experiment out of n>3 performed. p<0.01 for all treatment groups compared to control Jurkat cells (by Student's t-test).

Since our results indicated that 22bpCy3 transfer was independent of mature IS formation, we next asked whether cell-cell contact by itself was vital for miR intercellular transfer. To test this question, we physically separated the donor 22bpCy3 transfected BC-1 cells from the acceptor Jurkat cells during the co-culture period, by means of a transwell membrane (0.4μm pore size). Twenty four hours later, transfer of 22bpCy3 into Jurkat cells was determined by FACS. We found that synthetic miR spreading from BC-1 to Jurkat cells was largely contact-independent. However, at a 1:1 cell ratio, synthetic miRs spreading was more efficient in the conventional versus transwell membrane setup. Upon increasing the donor to acceptor cell ratio we observed increased contact-independent transfer of synthetic miRs from BC-1 to T cells (Figure 4A).

These results suggested that secretion of the synthetic miRs by BC-1 is an important mechanism for this transfer, and that the concentration of secreted miRs in the microenvironment of the T cells is probably a rate limiting factor. To further address this assumption, we transfected an excessive number of BC-1 cells with 22bpCy3, washed them after overnight incubation and 48hrs post transfection collected the culture supernatants. To effectively remove all cells and cellular debris we filtered the supernatants using a 0.2μm-filter. Thereafter, we cultured Jurkat cells in these 0.2μm-filterered supernatant, and by FACS determined that Jurkat cells acquired a significant quantity of labeled synthetic miRs from the BC-1-derived supernatants (Figure 4A, lower right histogram).

Next, we formally validated that miR-K12-11 transfer follows a similar scheme as 22bpCy3 transfer. Thus, we repeated the above experiments with non-transfected BC-1 cells, and at the end of the co-culture period analyzed the levels of miR-K12-11 in acceptor Jurkat cells by qRTPCR. We found that miR-K12-11 was indeed acquired by the Jurkat cells after being secreted from BC-1 cells in the upper chamber of the transwell, displaying a positive correlation between donor BC-1 to Jurkat cell ratio and miR-K12-11 intercellular spreading (Figure 4B).

To exclude that EBV co-infection of BC-1 plays a key role in the contact-independent spreading of miR-K12-11, we tested whether miR-K12-11 can spread from BCBL-1 lymphoma (infected only with KSHV) to Jurkat cells. Thus, we co-cultured BCBL-1 and Jurkat cells physically separated by means of 0.4μm pore size transwell membrane or incubated Jurkat cells with 0.2μm-filtered BCBL-1 supernatants. At the end of culturing, we determined the relative quantity of miR-K12-11 in Jurkat cells by qRTPCR. Similar to our finding with BC-1, we found that miR-K12-11 effectively transferred to Jurkat cells (Supplementary Figure 1). Analogous to the BC-1 data sets, we observed a positive correlation between miR-K12-11 cell-contact independent spreading and the donor BCBL-1 to acceptor T cell ratio.

Taken together, these results suggest that the secretory pathway is a major player in miR-K12-11 spreading, regardless of EBV co-infection, and that miR concentration in the environment is likely a rate limiting factor, while cell contact enhances the efficiency of miR transport.

### Mobile Cy3-miRs are RNase resistant and enter the cytoplasm of adopting Jurkat T cells

**Figure 5:**
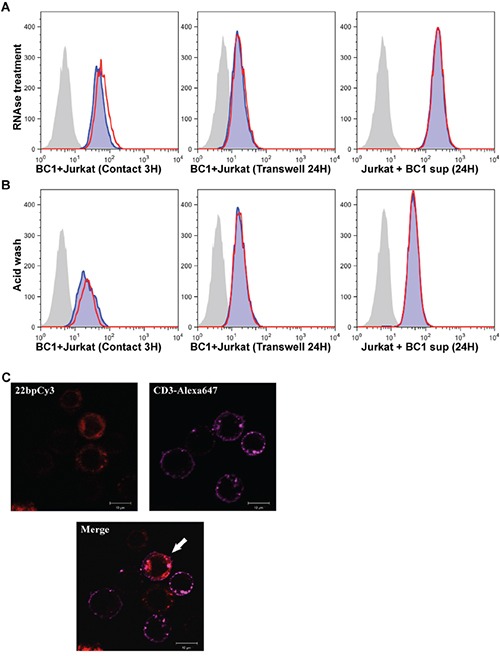
Mobile synthetic miRs are RNase resistant and enter the cytoplasm of adopting cells **A.** Jurkat cells were co-cultured for 3 hours in contact with 22bpCy3-BC-1 cells (left panel), or for 24 hours with 22bpCy3-BC-1 cells separated by transwell membrane (middle panel) or in filtered 22bpCy3-BC-1 culture derived supernatant (right panel). The latter cultures were treated with RNAse A+T mix (red line) or left untreated (blue shade). The grey shaded histogram represent background fluorescence of control Jurkat cells. **B.** In parallel, at the end of culturing counterpart cells were treated with acid wash (red line) or left untreated (blue shade). Results are typical for similar data obtained in >3 experiments. **C.** Confocal imaging of 22bpCy3 transfected BC-1 cells and Jurkat cells following 3 hours of co-culturing. The cells were fixed and stained with anti-CD3 Alexa 647-conjugated mAbs (far-red), and then subjected to confocal microscopy. Shown are images obtained from the red (Cy3) and far red (Alexa 647) channels and the merged image. The merged image shows that 22bpCy3 molecules localize to the cytoplasm of the CD3-labeled Jurkat cells (marked with a white arrow). Scale bars represent 10μm and images are from representative experiments out of 3 performed.

We next asked whether the secreted miRs were protected from RNAse activity. Since, our previous experiments show that 22bpCy3 can be applied instead of miR-K12-11 to assess intercellular spreading, we co-cultured 22bpCy3 transfected BC-1 cells and Jurkat cells as described above. Subsequently, we treated the various co-cultures and post-filtration supernatants with RNAse A/T1 cocktail and then analyzed the transfer of 22bpCy3. The FACS analysis demonstrated that RNAse treatment had no significant effect on the transfer and uptake of these miRs (Figure 5A). In addition we tested whether the Cy3-labeled miRs localized to the plasma membrane (PM) or to the cytoplasm of Jurkat cells as another indication of their biological role after entering other cells. To address this topic we first performed acid washing to bleach any Cy3 signal derived from the outer leaflet of the PM. The FACS analysis showed that acid washing had negligible effects on 22bpCy3 signal from Jurkat cells (Figure 5B). To further address the cellular localization of the mobile miRs, we imaged the cells after three hours of co-culturing. Prior to analysis the cells were immunostained with Alexa647-conjugated (far red) anti-CD3 mAbs to mark Jurkat cells, and then imaged by scanning confocal laser microscopy. We found that while the CD3 staining localized to the PM of Jurkat cells, the acquired Cy3-labeled miRs localized primarily to the cytoplasm (Figure 5C).

### Spreading of miR-K12-11 is independent of the classical exosome secretion pathway

**Figure 6:**
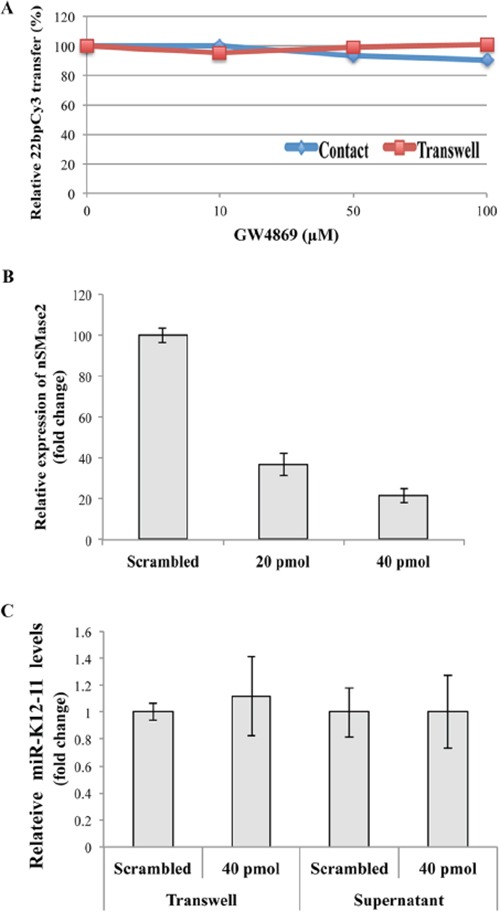
miR-K12-11 intercellular spreading is not dependent on the conventional exosomal secretion pathway **A.** Jurkat cells and 22bpCy3-BC-1 cells were treated beforehand with increasing concentrations of the GW4869 for 1 hour, and then co-cultured in the presence of the compound, either for 3 hours in contact or for 24 hours separated by transwell membrane. The acquisition of 22bpCy3 was determined by FACS. Graphs depict relative levels of mobile 22bpCy3Cy3 acquired by Jurkat cells, as a function of the concentration of the compound. **B.** BC-1 cells were transfected by electroporation with siRNA directed against nSMase2 and control scrambled siRNA. 48 hours later the relative (%) expression of nSMase compared to control transfection was analyzed by qRTPCR. **C.** Jurkat cells were cultured for 24 hours with BC-1 in a transwell system orwith BC-1 filtered supernatants. Bars represent fold-change of mir-K12-11 content in acceptor Jurkat cells transfected with either siRNA against nSMase2 or scrambled siRNA, the data calculation included normalization to a non-transfected Jurkat reference sample (mean ± S.E.M).

In recent years it has been suggested that cellular miRs can be carried by exosomes protected from degradation in the extracellular environment [8, 10, 18]. Classical exosomes are derived from multivesicular bodies and their release is triggered by sphingolipid ceramide [22]. To examine whether the intercellular transfer of miRs is mediated by this secretory pathway, we pre-treated the donor 22bpCy3 transfected BC-1 cells for 24 hours with the specific nSMmase inhibitor, GW4869, known to inhibit the classical exosome secretion pathway. Subsequently, the treated BC-1 cells were co-cultured with Jurkat cells for 3 hours or for 24 hours in a conventional or transwell membrane co-culture setups, respectively. The FACS analysis at culture end revealed that GW4869 did not reduce the transfer of 22bpCy3 either in the standard or in the transwell co-culture setup (Figure 6A).

Next, we determined that transfecting BC-1 cells with 40pmol of nSMase specific siRNA effectively reduced nSMase transcription by ~80% (Figure 6B). To answer whether nSMase2 knock-down inhibits the spreading of miR-K12-11, we transfected BC-1 cells with 40pmol nSMase-siRNA, and co-cultured them with Jurkat cells separated by a transwell membrane or in filtered supernatants of BC-1 cells. Twenty four hours later we analyzed the relative quantity of miR-K12-11 in the various Jurkat cell groups. We found that nSMase2 knock-down did not reduce the intercellular spreading of miR-K12-11 when compared to scrambled siRNA treated BC-1 cells (Figure 6C).

### Developing a biosensor system to detect miR-K12-11 action on target transcripts

**Figure 7:**
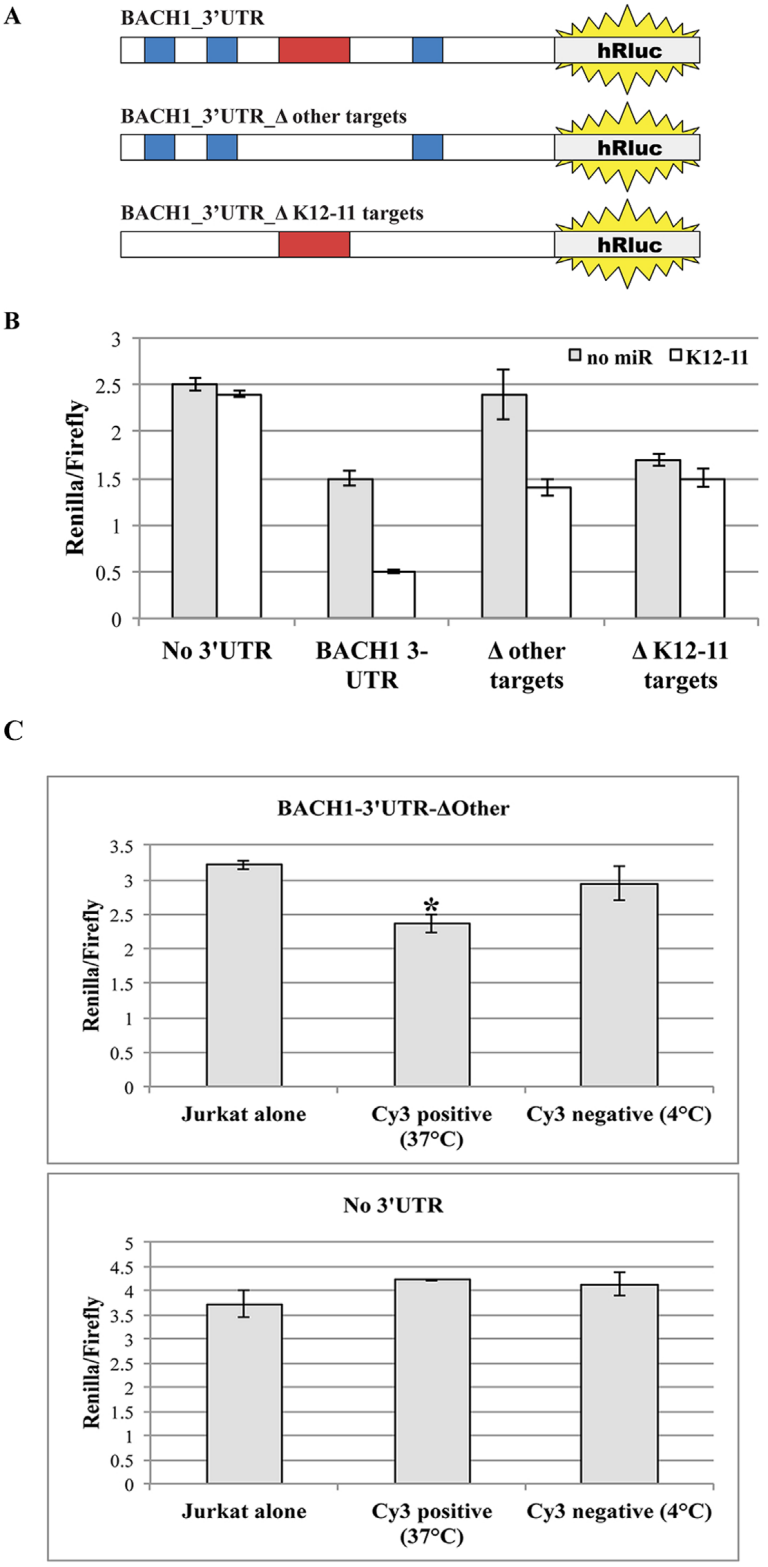
Mobile miR-K12-11 binds within acceptor T cells its canonical target BACH1-3′UTR **A.** Schematic representation of the different 3′UTR BACH1 constructs with the various mutations within miR recognition sites. **B.** BC-1 cells were transfected with the indicated reporter constructs. Each construct was co-transfected with or without synthetic miR-K12-11. Luciferase activity was assayed in triplicates 48 hours after transfection. **C.** Jurkat cells were transfected with the biosensor for miR-K12-11 activity, psiCHECK2-BACH1-3′UTR-ΔOther, or the empty control vector. Next, the transfected Jurkat cells were co-cultured with 22bpCy3-BC-1 cells for 3 hours. The 22bpCy3 served as a marker of miR transfer during the FACS sorting procedure. Cy3^neg^ and Cy3^pos^ Jurkat cells were sorted out from co-cultures maintained either at 37°C or 4°C. The sorted Jurkat cells were cultured for additional 48 hours to permit efficient repression of target mRNA translation. Subsequently the Jurkat cells were lysed and the inhibitory effect of mobile miR-K12-11 was determined by a dual luciferase assay in triplicates. Bars represent Renilla luciferase activity normalized to firefly luciferase activity (mean ± S.E.M). Data are from a typical experiment out of > 3 performed, *p<0.05 by Student's *t*-test.

The 3′UTR of transcription factor BACH1 is an experimentally validated target of both host miR-155 and its viral orthologue miR-K12-11 [11, 14]. As previously described by Gottwein et al., to directly detect the target binding activity of miR-K12-11, we cloned the 3′UTR sequence of BACH1 3′ to the hRluc ORF within the psiCHECK2 vector (Figure 7A). This enabled us to measure the effect of miR binding to 3′UTR-BACH1 on the translation efficiency of hRluc. Subsequently, this vector and the control empty psiCHECK2 vector were transfected into various Jurkat T cell groups with synthetic miR-K12-11 or alone. Forty-eight hours post transfection the cells were lysed and the effect of miR-K12-11 on hRluc luciferase activity was analyzed. We observed a strong inhibitory effect in the presence of miR-K12-11, but also a moderate decrease in relative hRluc activity in negative control Jurkat cells (Figure 7B). Our interpretation of the latter findings was that the 3′UTR of BACH1 may contain additional regulatory regions that are targeted by other endogenous cellular miRs expressed in Jurkat cells.

By bioinformatics analysis using TargetScan (http://www.targetscan.org), we found that the 3′UTR of BACH1 contains three binding sites for miR-K12-11 and at least four more binding sites for other human miRs, as follows: miR-142, miR-196, miR-292 and Let-7. By site-directed mutagenesis, we removed the binding sites for the other miRs (BACH1-3′UTR-Δ_Other_) and transfected Jurkat cells with this modified plasmid vector. We now found that without co-transfection of miR-K12-11 no significant changes were observed in hRluc activity as compare to counterpart Jurkat cells transfected with the empty psiCHECK2 vector (Figure 7B). Importantly, in Jurkat cells co-transfected with miR-K12-11 we indeed found a moderate (~33%) but significant decrease in hRluc activity. To better address the specificity of our miR-K12-11 biosensor system, we also removed the three putative binding sites of miR-K12-11 (BACH1-3′UTR-Δ_K12-11_). Following the co-transfection of the latter vector, we found moderate but significant down regulation of luciferase activity (~35%), as compare to the empty vector, indicating transcript targeting by other cellular miRs. However, we did not observe any significant changes in luciferase activity following co-transfection with synthetic miR-K12-11. These data confirm that the biosensor with BACH1-3′UTR-Δ_Other_ sequence cloned 3′ to hRluc ORF detects miR-K12-11 target binding activity with high specificity.

### Non-cell-autonomous miR-K12-11 is active and has biological function in the acceptor T cells

To determine whether the transferred miR-K12-11 is indeed active in the acceptor T cells, we co-cultured 22bpCy3 transfected donor BC-1 cells and Jurkat cells transfected with the psiCHECK2-BACH1-3′UTR-Δ_Other_ biosensor construct. At the end of co-culturing we sorted out with a FACSAria instrument a highly pure population of 22bpCy3^pos^ CD3^+^ Jurkat cells and cultured them alone for additional 48 hours to ensure optimal inhibition of target mRNA transcription. We found that in Jurkat cells the acquisition of 22bpCy3 was indeed associated with a statistically significant moderate inhibition (~25%) of hRluc activity compared to relevant control Jurkat cells (Figure 7C, upper graph). In addition, we repeated these experiments with Jurkat cells transfected with the empty psiCHECK2 vector, and as predicted we did not detect significant inhibition of relative hRluc activity following co-culturing with BC-1 cells (Figure 7C, lower graph). These results demonstrate that the transferred oncogenic miR-K12-11 is active and directly binds its target sequence in acceptor T cells.

**Figure 8:**
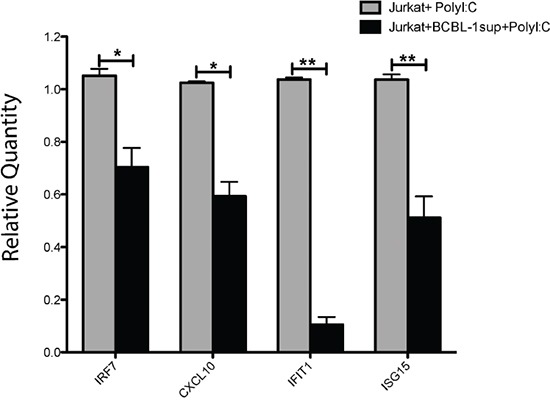
miR-K12-11 containing BCBL-1 supernatants reduce the TLR-3 and IKKε-dependent induction of ISGs Jurkat cells were cultured for 24 hours in medium containing 0.2μm-filtered BCBL-1 supernatants (50% per volume) or control medium. Subsequently, we stimulated the cells with the 5μg/ml of the synthetic TLR-3 ligand poly (I:C). Twenty four hours after stimulation, we purified total RNA from the cultures and determined by the SYBR Green qRTPCR Kit from Applied Biosystems and specific primers the relative quantity of mRNA transcripts of ISG15, CXCL10, IFIT1 and IRF7, as recommended by the manufacturer. Bars represent relative quantity (mean ± S.E.M, n=3) of the various mRNAs in treated (+BCBL-1sup) and untreated Jurkat cultures, calculated using the 2^−ΔΔCt^ method normalized to 18S rRNA.

To demonstrate a biological function of transferred KSHV miRs we focused on a well-described target gene of both miR-K12-11 and *hsa*-mir-155, the gene IKBKE/IKKε (a non-canonical IκB kinase homologue). Targeting IKKε is of particular importance, as this kinase is vital for the innate anti-viral response downstream of the dsRNA sensor TLR-3. Moreover, Liang et al. [3] reported that the high miR-K12-11 expression in BCBL-1 was coupled with a decrease in endogenous IKKε levels and in the ensuing transcription of typical IFN stimulated genes (ISGs). To validate that the transferred miR-K12-11 has a similar biological function in the acceptor cells, we cultured Jurkat cells in 0.2μm-filtered BCBL-1 supernatants or control medium for 24 hours. Next, we stimulated the cells for additional 24 hours with poly(I:C); a synthetic TLR-3 ligand and viral dsRNA mimetic. At the end of culture, we determined by qRTPCR the relative induction of four typical ISGs (ISG15, CXCL10, IFIT1 and IRF7). We found that overnight incubation in BCBL-1 supernatants was associated with significant inhibition of the poly (I:C) induced transcription of the four ISGs, with almost complete repression of the induction of IFIT1 (Figure 8). Thus, our data demonstrate at least one biological function of the transferred miR-K12-11, the inhibition of type-I interferons response to viral dsRNAs.

## DISCUSSION

The role of the immune system in controlling the development of virus-associated cancers is now well established [23]. A distinct features of both EBV and KSHV is that they express multiple miRs that are vital for viral latency and oncogenic transformation [11, 14, 24, 25]. In this work we show, for the first time that the KSHV-encoded oncogenic miR-K12-11 transfers from KSHV-infected lymphoma lines to uninfected T cells during short co-culturing. We also demonstrate that miR-K12-11 is biologically active in the acceptor T cells: binding its target sequence to inhibit reporter gene translation and suppressing the IKKε-dependent innate response to foreign dsRNAs.

Our group was the first to report that EBV-encoded miRs can spread among lymphocytes *in vitro* during co-culture [9]. For these studies we developed stringent FACS based methodologies to identify and sort-out pure T cells from the co-cultures, while purging with high accuracy B–T cell-fusion events. For example, we also used EBV-infected B721.221 cells engineered to express GFP to prove that B–T cell-fusion does not account for the transfer of the EBV-encoded BHRF-1-2 miR from infected B to uninfected T cells during short co-culturing of 1.5 hours [9]. This discovery was further confirmed by Pegtel et al. that detected EBV-derived miRs, but not viral DNA, in circulating T cells of subjects with a history of EBV infection [10]. Likewise, Masters and colleagues showed that EBV-encoded miRs can transfer *in vitro* to non-infected Thp-1 cells to inhibit the NLRP3 inflammasome in acceptor cells [26].

The working hypothesis that guided our present work was that intercellular spreading of virus-encoded miRs is another mechanism that γ-herpesviruses exploit to promote immune evasion. We focused on miR-K12-11 that is the orthologue of the oncomiR, *hsa*-miR-155. To address this notion, we initially used a FACS cell sorting based approach, previously established by us [9], to probe in real time the co-transfer of synthetic scrambled Cy3-conjugated miRs and miR-K12-11 from BC-1 to Jurkat cells. Thus, by physically sorting out 22bpCy3^+^ CD3^+^ singlet Jurkat cells after short co-culturing with BC-1 cells, we could determine unequivocally that miR-K12-11 transferred from BC-1 to Jurkat cells. In parallel, by confocal microscopy we determined that the mobile Cy3-labeled synthetic miRs, indeed, localized to the cytoplasm of the acceptor Jurkat cells, the normal site of interaction between endogenous miRs and their target mRNAs.

Although hypothetically possible, we reason that it is very unlikely that during three hours of co-culturing effective transmission of KSHV DNA to T cells with de novo transcription and biogenesis of miR-K12-11 accounts for a significant portion of miR-K12-11 detected in acceptor T cells. Moreover, such a theoretical scenario, can hardly explain the observed strong positive correlation between the spreading of 22bpCy3 and miR-K12-11.

Our next set of experiments was designed to address the cellular mechanism(s) involved in the spread of viral miRs between B and T lymphocytes. In this regard, we found that this transfer does not require the formation of a mature IS, as treatments with LatB, EDTA or blocking anti-LFA1 mAbs had only a minimal effect on miR spreading to Jurkat cells. Although, as previously shown [9, 18], direct contact between B and T cells accelerated the rate of intercellular miR transfer, it was not obligatory in our experimental system. The co-transfer of miR-K12-11 and 22bpCy3 occurred even when the donor and acceptor cells were separated by a transwell membrane. Moreover, we could determine that miR-K12-11 is indeed secreted into the media, by showing that Jurkat cells cultured in 0.2μm-filtered BC-1 or BCBL-1 supernatants acquired the viral oncomiR efficiently. The Cy3-labeled miRs that transferred to T cells were both RNase and acid wash resistant, indicating that they were transported by extracellular vesicles and/or in complex with protective chaperon proteins. These data together with previous reports [7, 9, 10, 18, 26–28] lead us to conclude that phagocytosis or virus-specific CTL responses do not contribute significantly to the transport of miRs across cell boundaries.

Intercellular communication is an essential hallmark of multicellular organisms. Cells release into the extracellular environment diverse types of vesicles, primarily exosomes and micro vesicles (MVs), derived from endosomal and plasma membrane, respectively [29]. To address the contribution of the classical exosome secretion pathway to the intercellular transfer of viral miRs in our system, we focused on silencing neutral sphingomyelinase-2 (nSMase2) that regulates the biosynthesis of ceramide and has established roles in classical exosome formation [22]. RNAi mediated silencing of nSMase2 or its pharmacological inhibition with GW4869 have been shown to significantly reduce the secretion of exosomes by various laboratories [18, 30, 31]. Nevertheless, in our co-culture system, neither silencing nSMase2 transcription in BC-1 cells nor its inhibition with GW4869 produced a significant inhibition of the intercellular spreading of miR-K12-11.

In this regard, Arroyo et al. determined that a significant number of circulating extracellular human miRs are in complex with Ago2 and are largely excluded from exosomes [32]. A following paper by Turchinovich et al. confirmed these observations also in supernatants of various human cell lines [33]. Others have reported that extracellular miRs can also be transported by high-density lipoprotein particles [34]. More recently, Pegtel and colleagues published a comprehensive study that addresses the underlying mechanisms dictating miR sorting into exosomes. They discovered that endogenous miRs display a nonrandom distribution between the cellular and exosomal compartments, and that 3′-uridylation of miR species correlated with their enhanced sorting into exosomes. Analysis of the distribution of EBV-encoded miRs in infected B cell lines revealed that viral miRs were largely excluded from exosomes. Thus, the above cited reports and our current findings, point out that the γ-herpesvirus-encoded miR-K12-11 is not transported via classical exosomes. However, as the mechanisms for generation of exosomes and MVs are complex and incompletely defined, our data do not categorically exclude the possibility that other types of MVs transport miR-K12-11 and other γ-herpesvirus-encoded miRs.

Finally, we determined that the non-cell-autonomous miR-K12-11 was biologically active within the acceptor Jurkat cells. Using a validated biosensor system [11], we determined that the transferred miR-K12-11 binds to its canonical target sequence in the 3′UTR of BACH1, consequently reducing the translation of the upstream reporter hRluc gene. Importantly, focusing on a well-described target of miR-K12-11 and *hsa*-mir-155, the non-canonical IκB kinase IKKε, we show a correlation between miR-K12-11 transfer into Jurkat cells and a reduced type-I interferons response to foreign dsRNAs. Targeting IKKε is of particular importance, as this kinase is vital for the innate response downstream of cytosolic sensors of viral RNAs such as TLR-3 [3, 35, 36]. Phosphorylation of IKKε leads to activation of IRF3 transcriptional activity and the induction of IFNβ and various IFN-stimulated genes (ISGs). For example, Liang et al. [3] reported that BCBL-1 cells express high levels of miR-K12-11 (but low levels of miR-155) that was coupled with a decrease both in IKKε levels and in IRF3-dependent transcription of typical ISGs. Correspondingly, we found that incubation of Jurkat cells with miR-K12-11 containing supernatants correlated with reduced expression of four typical ISGs (ISG15, CXCL10, IFIT1 and IRF7) following the activation of the TLR-3/IKKε/IRF3 cascade by Poly (I:C). In this regard, CXCL10 is an important chemoattractant for effector CD8^+^ T cells [37] and thus it can be predicted that targeting the induction of this chemokine is an additional mechanism employed by KSHV for immune evasion.

The miRs encoded by KSHV and EBV generally do not contain similar seed sequences, but they share many target genes including the pro-apoptotic proteins BIM and PUMA [14, 38, 39]. To evade destruction by cytotoxic T and NK cells, KSHV- and EBV-encoded miRs target both the T cell chemokine CXCL-11 [40] and the ligand for Killer Activation Receptors MICB [24]. They also encode miRs that target IRAK1 and MyD88 to suppress signaling downstream of IL-1 and TLRs [3, 25, 41]. In light of our current findings, it is conceivable that KSHV/EBV dually infected B cells can transfer a large variety of bioactive virus-encoded miRs to T cells, producing a cumulative inhibitory effect on the anti-viral response.

In summary, we show for the first time that a KSHV-encoded miR with an established oncogenic potential spreads from infected lymphoma cells to neighboring T cells to reduce target gene translation and suppress the IKKε-dependent innate response to viral dsRNAs. Thus, describing a new non-cell-autonomous mechanism that γ-herpesviruses use to restrict host T cell-dependent immunity and potentially promote carcinogenesis.

## MATERIALS AND METHODS

### Antibodies and reagents

Allophycocyanin (APC)-conjugated mouse mAbs directed against human T cell receptor (CD3) were obtained from eBioscience Inc. (San Diego, CA). The following reagents were purchased from Sigma-Aldrich Co. LLC: Latrunculin B (LatB), PP2 (Src-family tyrosine kinase Inhibitor), Phorbol 12-myristate 13-acetate (PMA), GW4869 (non-competitive neutral sphingomyelinases inhibitor), and Polyinosinic-Polycytidylic acid [poly (I:C)]. The Cy3-labeled Pre-miR™ Negative Control #1, a “scrambled” synthetic miR mimetic conjugated to the Cy3 flourochrome (22bpCy3) was obtained from Ambion (Thermo Fisher Scientific Inc.). Synthetic mature KSHV-miR-K12-11 (miR-K12-11) and a validated small interfering (si)RNA targeting human nSMase2 and a scrambled negative control siRNA were also obtained from Ambion.

### Cell lines

The human Jurkat T cell line was obtained from ATCC (American Type Culture Collection). The KSHV-infected BC-1 and BCBL-1 cell lines were a generous gift from Dr. Ronit Sarid (Bar-Ilan University, Israel). Cell lines were cultured in complete RPMI-1640 medium supplemented with 10% fetal bovine serum (FBS), 2 mM L-glutamine, 100 U/ml penicillin and 100 μg/ml streptomycin (all from Gibco, Carlsbad, CA), and maintained at 37°C in a humidified 5% CO_2_ incubator.

### Fluorescence-activated cell sorting (FACS)

For multi-parametric FACS analysis, cell samples were acquired and analyzed on a FACSCalibur™ using Cellquest™ software or alternatively on a FACSAria™ instrument using FACSDiva™ software (all from BD Biosciences). Data were collected from ~10,000 single-cell events. T cell singlet-cell events were distinguished from target cells by their by CD3 staining and their distinct FSC/SSC characteristics, as previously published [17, 42]. Final data analysis was performed using the Flow-Jo software (Tree Star, OR). All the cell-sorting experiments were performed, as previously described, on a FACSAria instrument [9]. Briefly, to obtain a single-cell suspension, cells were pretreated with EDTA and vortexed to dissociate cell-conjugates and then maintained at 4°C. To sort out only viable Jurkat-singlet cells we employed a stringent multiparametric gating strategy including FSC and SSC characteristics (pulse width, height, and area) together with CD3 labeling. For performing downstream analysis post-sort purity >95% for the relevant CD3^+^ singlet cell population was required.

### Microscopy

BC-1 cells 48 hours post-transfection with 22bpCy3 and Jurkat cells were co-cultured for 3 hours. Next, the cells were fixed in 2% paraformaldehyde, stained with anti-CD3-Alexa 647 mAbs (far-red), seeded onto slides and subjected to laser scanning confocal microscopy (Zeiss LSM510).

### Co-cultures and analysis of intercellular transfer

The KSHV-infected BC-1 and BCBL-1 cell lines were transfected with the various synthetic miRs/siRNAs by Amaxa electroporation (Program T-001), according to manufacturer's instructions. Twenty-four hours post transfection with the cells were thoroughly washed to remove surplus extracellular miRs/siRNAs prior to co-culturing. Next, to examine the intercellular co-transfer of 22bpCy3 and miR-K12-11, the BC-1 transfectants were distributed into U-bottom tubes (0.5 × 10^6^ cells per tube in 0.5 mL) to which T cells were added (0.5 × 10^6^ cells per tube in 0.5 mL) to obtain an effector to target ratio of 1:1. The culture tubes were centrifuged for 2 minutes at 1000 rpm to promote cell-conjugate formation and then co-cultured for 3 hours at 37°C. For FACS based analysis of 22bpCy3 transfer, the collected cells were suspended vigorously in 5 mM EDTA/PBS and kept on ice for 30 min to allow cell conjugates to dissociate. Next, the cells were immunostained with flourochrome-conjugated anti-CD3 mAbs to label T cells, washed and resuspended in 5 mM EDTA/PBS.

### RNA isolation and real time quantitative PCR (qRTPCR)

The mirVana miR isolation kit (Ambion, Thermo Fisher Scientific Inc.) was used to isolate total RNA per manufacturer's instructions. The quantification of miR-K12-11 was performed by qRTPCR, in triplicates, using a custom designed TaqMan® miR assay and analyzed on an ABI PRISM 7300 Sequence Detection system (all from Applied Biosystems, Thermo Fisher Scientific Inc.). The levels of miR-K12-11 were normalized, as customary, to a ubiquitous snRNA (RNU6B). The relative quantity of the mRNA of selected ISGs (ISG15, CXCL10, IFIT1 and IRF7) was determined by specific primers using the SYBR Green qRTPCR Kit (Applied Biosystems), as recommended by the manufacturer. All reactions were done in triplicates and normalized to 18S rRNA, and relative quantity was calculated using the 2^−ΔΔCt^ method.

### BACH1 3′UTR mutagenesis and cloning into the psiCHECK2 vector

BACH1 3′UTR was amplified from human genomic DNA by a specific set of primers (CCCTTGATTTCCTACCTCAGTG and CTTCGGCAGCCTCAAAAA) designed to include 3 putative hsa-miR-155 / KSHV-miR-K12-11 binding sites. These bioinformatics data were generated based on miRNA target prediction algorithms (http://www.targetscan.org); genomic sequence databases available from the UCSC genome browser (http://genome.ucsc.edu); and the Primer3 online tool (http://frodo.wi.mit.edu/primer3). Next, the amplified fragment was cloned into pGEM T-easy vector (Promega, Madison, WI, USA), verified by sequencing and compared to the original human genome sequence. pGEM-BACH1 3′UTR was digested using NotI restriction enzyme (New England Biolabs Ltd.) and cloned into the psiCHECK2 vector (Promega, Madison, WI, USA) into the multiple cloning region located 3′ to the synthetic Renilla luciferase (hRluc) gene. Thereafter, a clone where the BACH1 3′UTR was inserted in the correct orientation was selected for future *in vitro* studies. To reduce the effect of other miRs on hRluc expression, a small region in BACH1 3′UTR containing putative target sites for miR-142, miR-196, miR-292 and Let-7 was deleted using a specific set of primers (Supplementary Table 1) and Quickchange mutagenesis kit (Agilent Technologies Inc.). The mutation was verified by sequencing and compared to the normal genomic sequence. This latter vector (psiCHECK2-BACH1-3′UTR-ΔOther) was eventually used as the biosensor to specifically detect miR-K12-11 activity.

### Analyzing miR-K12-11 target binding activity by the dual-luciferase assay

To determine the miR-K12-11 binding activity, Jurkat cells were transfected with 500ng of a modified psiCHECK2 vector (Promega, Madison, WI, USA) containing a modified BACH1 3′UTR with or without 20pmol of the mature miR-K12-11 oligonucleotides using Amaxa pulse-program X-05. Forty-eight hours post transfection, the Jurkat cells were washed carefully with PBS, and lysates were assayed for luciferase activity in triplicates by the Dual-Luciferase Reporter Assay (Promega, Madison, WI, USA). *Renilla* luciferase activity was normalized to *Firefly* luciferase activity.

### Transwell assay

Jurkat cells were prevented from directly contacting BC-1 or BCBL-1 cells by a semi-permeable 0.4μm pore size transwell membrane (Costar). Briefly, 0.5 × 10^6^ Jurkat cells were placed in the lower chamber (in 1 mL of medium) and 0.5 × 10^6^ B lymphoma cells (in 0.5 mL of medium) were added to the upper compartment (in 12-well plates). The cells were incubated for 24 hours at 37°C. At the end of co-culturing, the cells were collected in 5 mM EDTA/PBS and analyzed for 22bpCy3 acquisition by Jurkat cells as described above.

### Acid wash and RNAse treatment

Cells were washed twice in PBS, resuspended and incubated for 4 min at 20°C in acidic citrate buffer (0.13 M citric acid and 0.06 M Na2HPO4 at pH 3.3). To determine whether the transferred miRs are protected from RNAse mediated degradation, the cell cultures were treated with RNAse A/T1 cocktail (Thermo scientific, St. Leon-Rot, Germany) during the co-culture period.

## SUPPLEMENTARY FIGURE AND TABLE


