# Supplementary material for: Viral oncomiR spreading between B and T cells is employed by Kaposi's sarcoma herpesvirus to induce non-cell-autonomous target gene regulation
Source: Oncotarget. 2016 May 26;7(27):41870–84. doi: 10.18632/oncotarget.9627 (PMC5173102; doi:10.18632/oncotarget.9627)
Supplement: Supplementary file 1 [file oncotarget-07-41870-s001.pdf]

Viral oncomiR spreading between B and T cells is employed by Kaposi's sarcoma herpesvirus to induce non-cell-autonomous target gene regulation

SUPPLEMENTARY FIGURE AND TABLE

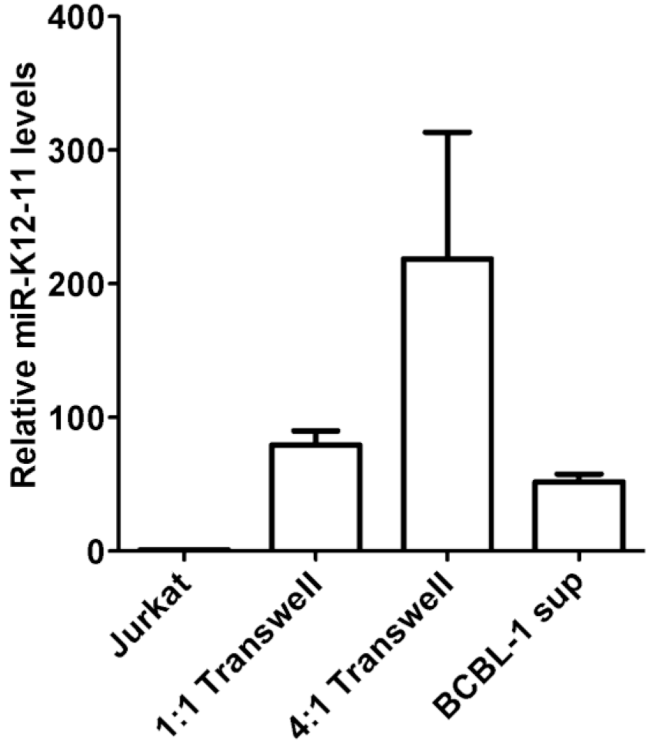

Supplementary Figure S1: BCBL-1 cells transfer virus-encoded mir-K12-11 to Jurkat T cells. A. Jurkat cells were co-cultured for 24 hours with BCBL-1 cells separated by transwell membrane (in donor-to-acceptor ratio of 1:1 or 4:1) or in BCBL-1 filtered supernatant. At the end of co-culturing in the transwell system, total RNA was isolated from the various cell cultures for qRTPCR analysis. Bar chart represents the fold-change of relative mir-K12-11 levels (mean + S.E.M of duplicates) in the various Jurkat groups compared to control cells. Depicted is a typical experiment out of n=3 performed. $p<0.01$ for all treatment groups compared to control Jurkat cells (by Student's t-test).

Supplementary Table S1: List of the sequences of specific primers used to mutate the BACH1-3'UTR

Site of mutation	Sense primer	Anti-sense primer
miR-K12-11 #1	CTCTCTACCTATAAACAGTTTAGGGT TTCTATTAATGACACAG	CTGTGTCATTAATAGAAACCCTAAAC TGTTTATAGGTAGAGAG
miR-K12-11 #2	GGCCAAGTGTAATTTCTTAAAATTTCTT TAAATAGCCAGCATG	CATGCTGGCTATTTAAAGAAATTTTA AGAAATTACACTTGGCC
miR-K12-11 #3	GGGAAAGTTTTCTGTATATTGCATCACATTTAT GCCTATTTTAAC	GTTAAAATAGGCATAAATGTGATGCA ATATACAGAAAACTTTCCC
ΔOther miRs	GCATGTAATACAAGTAACTACCATATCTACAT GATTTTCAAGTTG	CAACTTGAAAATCATGTAGATATGGTA GTTACTTGTATTACATGC

To reduce the effect of activity of other miRs on hRluc expression, a small region in BACH1- 3'UTR containing putative target sites for miR-142, miR-196, miR-292 and Let-7 was deleted using a specific set of primers and Quickchange mutagenesis kit (Agilent Technologies Inc.). The mutation was verified by sequencing and compared to the normal genomic sequence. In addition, as an additional control we generated a vector where all the 3 putative target-sites for miR-K12-11 were removed from BACH1 3'UTR.
